# Development and Preliminary Validation of the Youth Leadership Potential Scale

**DOI:** 10.3389/fpsyg.2019.02310

**Published:** 2019-10-17

**Authors:** Yue Yuan, Qing Chen, Xiaomin Sun, Zhenzhen Liu, Gang Xue, Di Yang

**Affiliations:** ^1^Beijing Key Laboratory of Applied Experimental Psychology, National Demonstration Center for Experimental Psychology Education (Beijing Normal University), Faculty of Psychology, Beijing Normal University, Beijing, China; ^2^Yunnan Provincial Natural Gas Co., Ltd., Kunming, China; ^3^Department of Public Administration, Chinese Academy of Governance, Beijing, China; ^4^Department of Human Capital Management, New York University, New York, NY, United States

**Keywords:** the Youth Leadership Potential Scale, development theory of leadership, scale development, leadership potential, exploratory structural equation modeling

## Abstract

To address the need for a valid and reliable scale of youth leadership potential based on the development theory of leadership, the current study developed the Youth Leadership Potential Scale (YLPS) and investigated its factor structure and psychometric properties in a sample of 696 students (grades 7–9) in China. Exploratory structural equation modeling (ESEM) identified a five-factor solution comprising leadership information, leadership attitude, communication skills, decision-making skills, and stress management skills. ESEM within confirmatory factor analysis demonstrated an adequate fit for this structure. The scale showed good composite reliability and measurement invariance across different gender and grade/age groups. The scale also showed sufficient concurrent validity with the Coping Self-Efficacy Scale, the Chinese Roets Rating Scale for Leadership, and the Leadership Skills Inventory. Furthermore, criterion-related validity was supported by the relationship between YLPS scores and the length of student leadership positions. The results suggest that the YLPS is a valid and pragmatic measure for assessing youth leadership potential. The current study is the first to develop a youth leadership potential scale based on the development theory of leadership.

## Introduction

Leadership is like a “catalyst” that enables all other business aspects to work together. Leadership is essential to the survival and success of an organization (Antonakis and Day, 2017) because it facilitates the maximization of organizational efficiency and the achievement of organizational goals (Higgs and Aitken, 2003). As Chambers et al. (1998) suggested, the effectiveness of a leader is a major determinant of organizational performance. One limitation of the extant literature on leadership development is that it heavily focuses on leadership development and practice in adulthood (Chan, 2000), while relatively little attention has been devoted to youth (Murphy and Johnson, 2011).

Early experiences play a critical role in the development of leadership (Riggio and Mumford, 2011) by serving as the foundation for leadership development in adulthood (Karagianni and Montgomery, 2018) and increasing the possibility that an individual will grow up to become a leader. For example, research suggests that students with leadership experience in high school are more likely to become managers in adulthood (Kuhn and Weinberger, 2005). Early points in life are a sensitive period for the development of many leadership-related personality traits and skills (Riggio and Mumford, 2011) that are developed more easily and rapidly early on. Although early development of these traits and skills does not guarantee successful development in adulthood, it sets the stage for future development to occur (Murphy and Johnson, 2011). In addition, early experience in leadership facilitates the developmental process through the self-reinforcing mechanism (Murphy and Johnson, 2011). That is, the more leadership capacity individuals acquire in their early experiences, the more motived they are to engage in further leadership activities and vice versa. From this point of view, early leadership experiences are likely to be a trigger of the leadership development process.

It was not until recently that some researchers began to investigate “the seeds of leadership” (Day, 2011; Gottfried et al., 2011; Murphy and Johnson, 2011; Reichard et al., 2011). Youth leadership is inherently future oriented. An important distinction between youth and adult leadership development is that the former tends to plan for future leadership (Redmond and Dolan, 2016). That is, its focus is on the need to identify, cultivate and facilitate the development of youth so that they can become effective leaders later (MacNeil, 2006). Thus, gauging young people’s leadership potential is a critical starting point that enables them to understand their strong and weak points in leadership development. As a result, they can intentionally choose different learning opportunities and activities to address their deficiencies accordingly.

Evaluations of leadership can also equip educators with clearer goals and purposes in designing and customizing leadership training programs. Although there is a dearth of research and theory on youth leadership, programs targeted at youth leadership development have been around for many years. Such programs include extracurricular activities (Hancock et al., 2012), community programs run by youth (Larson et al., 2005), and camps (Thurber et al., 2007). The key problem of these programs is that most lack support from theory on leadership development (Seemiller, 2018). As a result, these programs are generally collections of interesting leadership activities without the intentional goal of development. The evaluation of leadership potential among youth could be used to develop such programs so that adolescents can customize their curricula according to what they need most to be future leaders.

The word “potential” means “existing in possibility; capable of development into actuality; a power or quality that has not yet come forth but may emerge and develop” (Tiffan, 2009). It is assumed that potential is a dynamic and not an end state (Silzer and Church, 2010). In organizations, potential “refers to the possibility that individuals can become something more than what they currently are. It implies further growth and development to reach some desired end state” (Silzer and Church, 2009). Accordingly, leadership potential is defined as the possibility that an individual could develop and acquire those leadership competencies that are highly challenging. In general, individuals with high leadership potential possess some cognitive, emotional and behavioral growth factors that make it possible for them to grow into great leadership (Silzer and Church, 2009).

The extant literature lacks effective tools with which to evaluate youth leadership potential. Table 1 shows the commonly used youth leadership scales. As observed, these scales are mainly limited to the assessment of certain specific leadership skills. To facilitate research and practice in youth leadership development, the aim of the current study is to develop and validate the Youth Leadership Potential Scale (YLPS) based on the development theory of leadership.

**TABLE 1:** A brief summary of youth leadership scales in extant literature.

**Scales**	**Author(s)**	**Year**	**Sample**	**Respondent:**	**Dimensions**	**Number of**
				**self/other**		**items**
Leadership Ability Evaluation (LAE)	Cassel, Stancik	1982	Grade 9 to adult	Self	Four leadership decision styles: laissez faire, democratic-cooperative, autocratic-submissive, autocratic-aggressive	50
Leadership Skills Inventory (LSI)	Karnes, Chauvin	1985	Grade 4–12	Self	Fundamentals of leadership; written communication skills, speech communication skills, values clarification, decision-making skills, group dynamics skills, problem-solving skills, personal development skills, planning skills	125
Leadership: A Skill and Behavior	Sisk	1987	–	Self	Positive self-concept, communication skills, decision-making skills, problem-solving skills, group dynamics skills, organizing, planning skills, implementing skills, and discerning opportunities	33
Eby Gifted Behavior Index – leadership subscale	Eby	1983	Grade K-12	Teachers	Perceptiveness, active interaction with the environment, reflectiveness, persistence, independence, goal orientation, originality, productivity, self-evaluation, effective communication of ideas	20
Roets Rating Scale for Leadership (RRSL)	Roets	1997	Grade 5–12	Self	Task orientation, leadership self-efficacy, and leadership flexibility	26
Leadership Skill Scale	Ogurlu, Emir	2013	Grade 6–8	Self	Problem solving, group dynamics, timidity, goal setting, empathy, leadership, anger management, perseverance, creativity, and speech communication	41

### The Development Theory of Leadership

The development theory of leadership proposed by Linden and Fertman (1998) believes that every adolescent possesses leadership to an extent and demonstrates their leadership abilities in subtle ways in their family life, school activities and interactions with neighbors in their communities. The theory proposes that the development of youth leadership fits into five dimensions: (1) leadership knowledge and information, (2) leadership attitude, will, and desire, (3) communication skills, (4) decision-making skills, and (5) stress management skills (Ricketts and Rudd, 2002). These five dimensions include the cognitive, emotional and behavioral aspects of youth leadership development (Linden and Fertman, 1998) and can be used as indicators of youth leadership potential.

The *leadership knowledge and information* dimension refers to the knowledge that adolescents must have about leadership before they can act as leaders (Ricketts and Rudd, 2002). Appropriate knowledge and information about leadership are helpful in making the abstract and complex leadership concept more concrete and operational (Ricketts and Rudd, 2002). The *leadership attitude, will, and desire* dimension refers to adolescents’ leadership inclination, motivation, and interest (Lord and Hall, 2005). Because there are unavoidable challenges and frustrations throughout the process of leadership development, highly motivated adolescents are more likely to proactively learn from their experiences (Chan and Drasgow, 2001).

According to Linden and Fertman (1998), high-potential young people develop an array of leadership skills during their development process; these skills include communication, decision-making, and stress management. *Communication skills* represent the ability to present ideas to and exchange information with others. Researchers believe that communication is the basic mechanism by which leaders inspire or influence others (Boies et al., 2015). *Decision-making skills* represent the ability to make good choices with available information. Leaders must make a multitude of important decisions, and leaders’ performance strongly depends on how effective they are in solving complex problems arising in organizations (Mumford et al., 2000). Thus, decision-making skills are crucial for the effectiveness and success of leaders. *Stress management skills* represent the ability to effectively respond to and manage stressful situations. Leaders are often under considerable stress (Day et al., 2004), and stress consumes a great deal of cognitive and emotional resources, making it difficult for leaders to function effectively (Harms et al., 2017). Thus, stress management skills are a key to effective leadership. As shown in Table 1, these three leadership skills are embedded in many previous youth leadership scales.

The development theory of leadership was developed based on previous research findings (Fertman and Long, 1990; Fertman and Chubb, 1992; Wald and Pringle, 1995; and Long et al., 1996). Later researchers provided supporting evidence for the theory. For example, the theory is consistent with the experiential learning theory of Kolb (2014), which is a holistic integrative perspective on learning that combines experience, perception, cognition, and behavior. Ricketts and Rudd (2002) constructed a conceptual model after performing a meta-analysis of the literature on adolescent leadership development. Their model also proposed that youth leadership development includes five dimensions. Four of the five dimensions composing the model are consistent with the development theory. In sum, the theory provides a consistent framework for assessing, monitoring and evaluating the development of youth leadership. For example, based on this theory, Anderson (2009) examined students’ perceptions of the preferences for leadership development, and Bruce and Stephens (2017) suggested a practical framework to facilitate student leadership development in various kinds of clubs and student governance.

Using the development theory of leadership as the framework, the present study developed and preliminarily validated the YLPS. Although the theory proposed by Linden and Fertman (1998) is used as the guideline for many research and practical programs (Turkay and Tirthali, 2010; Bruce and Stephens, 2017), the present study is the first to construct a youth leadership potential scale based on this theory.

## Materials and Methods

### Participants and Procedure

The cross-sectional study was conducted with a sample comprising 702 students from a middle school in Kunming, China. Data of six participants were removed because the proportion of missing values in their responses was higher than 10%. The remaining 696 participants formed the final dataset. Among these participants, 319 were males (45.8%), and 377 were females (54.2%). They were in seventh (20.1%), eighth (29.2%), and ninth grade (50.7%). In China, students in the same grade generally have limited variance in their age (the seventh grade is usually aged 12–13 years old, the eighth grade 13–14 years old, and the ninth grade 14–15 years old). In terms of parents’ highest educational degree obtained, 51 were primary school or lower (7.3%), 143 were middle school (20.5%), 176 were high school (25.3%), 122 were college (17.5%), 169 were university (24.3%), and 25 were master or higher (3.6%). Table 2 shows the detailed demographic information of the participants.

**TABLE 2:** Demographic information.

**Variables**	***n***	**%**
**Gender**		
Male	319	45.8
Female	377	54.2
**Grade**		
Seventh (12–13 years old)	140	20.1
Eighth (13–14 years old)	203	29.2
Ninth (14–15 years old)	353	50.7
**Parents’ highest educational degree obtained**		
Primary school or lower	51	7.3%
Middle school	143	20.5%
High school	176	25.3%
College	122	17.5%
University	169	24.3%
Master or higher	25	3.6%

The survey questionnaire consisted of a set of self-report measures and demographic questions. The measures were group-administered in classroom settings. Classroom teachers administered the survey with the assistance of a graduate student majoring in psychology. The aim of the study and participation guidelines were clearly stated in the title page of the survey. All questionnaire sets were completed anonymously. Students were also informed that they could quit the survey at any time during the process and the information provided would be kept strictly confidential and used solely for research purposes. Formal consent was obtained from the students before they started the survey. Each student received 10 Chinese Yuan for their participation. Debriefing information was provided at the end of the survey. The study was reviewed and approved by the Academic Ethics Committee at the first author’s institution before being conducted.

### Measures

*The YLPS* was developed to assess the leadership potential of adolescents. Before the item generation stage, semi-structured interviews were conducted to collect typical examples of the demonstration of the five dimensions of youth leadership potential. Specifically, 20 middle school teachers, 5 parents, and 16 middle school students from six different middle schools in China participated in the interviews. According to the descriptions of each dimension in Linden and Fertman (1998), the operational definitions of the five dimensions were provided to all interviewees. Specifically, the leadership information dimension refers to youths’ understanding of leaders and leadership, including leaders’ responsibilities, what leadership is and what leadership does. The leadership attitude dimension refers to youths’ thoughts and feelings toward being a leader. The communication skills dimension refers to the ability to understand other’s verbal and non-verbal message and effectively express one’s own ideas. The decision-making skills dimension refers to the ability to cautiously make good use of available information and come to a rational choice. The stress management skills dimension refers to the ability to effectively cope with and deal with stress in daily life.

Participants were asked to provide three cases in which they observed a student who demonstrated all or part of these dimensions. For teachers and parents, cases were limited to students they taught or children of their own. For students, cases could be about themselves or their classmates. Each interview lasted approximately 1 h. The interview was recorded with permission and was transcribed by professionals afterward, resulting in a document of 534475 words in total. Teachers and parents each received 100 Chinese Yuan for their participation. Students each received a gift at the end of the interview.

By combining the theoretical consideration and the rich examples collected in the interview stage, we initially generated a pool of 39 items covering the five dimensions of youth leadership potential. For those dimensions for which classic scales are available, some items were adopted from related studies and existing scales [e.g., Leadership Skills Inventory (LSI), Karnes et al., 1985; Roets Rating Scale for Leadership (RRSL), Roets, 1997; and Leadership Ability Evaluation, Cassell and Stancik, 1982]. A total of nine items were adopted from previous measures, and thirty items were self-developed.

Subsequently, a panel of two psychologists evaluated the initial pool of 39 items. One of the psychologists was an expert in adolescence and the other was a leadership researcher. All items were evaluated based on three criteria: item relevance and item specificity to the to-be-evaluated dimensions as well as item clarity. Globally, the experts’ appraisals were positive, and some items were revised according to their comments and suggestions. The revised 39 items formed the preliminary scale. All items were evaluated by participants on a 5-point Likert scale (from 1 = strongly disagree to 5 = strongly agree). Before formal data collection, a pilot test was administered to a few middle school students in seventh, eighth, and ninth grade to identify possible misunderstanding of the items. Small modifications were made accordingly.

*The Coping Self-Efficacy Scale (CSES)* was used to evaluate adolescents’ confidence in engaging in coping behaviors when faced with life challenges (Chesney et al., 2006). The scale includes 13 items assessing three dimensions: (a) problem-focused coping (e.g., “break an upsetting problem down into smaller parts”), (b) emotion-focused coping (e.g., “take your mind off negative thoughts”), and (c) social support (e.g., “get emotional support from friends and family”). In the current study, the CSES was translated into Chinese strictly following the back-translation procedure. All items (α = 0.850) were rated on a 10-point scale ranging from 1 (not at all likely) to 10 (extremely likely).

*The Roets Rating Scale for Leadership (RRSL)* is a self-report scale that evaluates the leadership characteristics of students (Roets, 1997). In the current study, we adopted the Chinese revision of the RRSL (CRRSL) developed by Chan (2000). It consists of 15 items with three dimensions: leadership self-efficacy (e.g., “have self-confidence”), leadership flexibility (e.g., “can understand others’ views”), and task orientation (e.g., “promote what is believed”). Participants were asked to rate the items (α = 0.900) on a 5-point scale from 1 (almost never) to 5 (almost always).

*The Leadership Skill Inventory (LSI)* was designed to assist students in analyzing the strength of their leadership skills (Edmunds, 1998). The Chinese version (LSI-C) developed by Wang et al. (2012) was used in the present study. The LSI-C assesses 5 leadership skills: teamwork (e.g., “I get along well with people around me”), self-understanding (e.g., “I think we should be responsible for our actions”), communication (e.g., “I am a good listener”), decision-making (e.g., “I will make decisions based on my previous experience”), and fundamentals of leadership (e.g., “I am respected by my peers”). Participants were asked to rate these 21 items (α = 0.890) using a 5-point scale ranging from 1 (strongly disagree) to 5 (strongly agree).

*The length of student leadership positions* was collected to estimate the criterion-related validity of the YLPS. It was measured by the question “How many years in total since primary school have you been a student leader?”

*Demographic variables* included gender, grade, and family SES. Participants’ gender was dummy coded, with females coded as 0 and males coded as 1. Grade was coded into three categories, with seventh grade coded as 1, eighth grade coded as 2, and ninth grade coded as 3. Family SES was calculated based on parents’ highest educational degree obtained, parents’ occupation, and family property (Yuan et al., 2009; Oecd, 2010).

### Data Analysis

First, we conducted missing values analysis and sample size adequacy estimation in the preliminary analyses to ensure that the data was suitable for further analysis. Then, we examined three aspects of the psychometric properties of the YLPS. (1) We conducted exploratory factor analysis (EFA) and exploratory structural equation modeling (ESEM) to examine the factorial validity of the scale. (2) We examined the composite reliability of subscales using McDonald’s (1970)ω. (3) We examined the concurrent and criterion-related validity of the scale. The preliminary analyses and EFA were conducted using SPSS 22.0 (IBM SPSS Inc., Chicago, IL, United States). Other data analyses were conducted using Mplus 7.11 (Muthén and Muthén, 2019). In particular, the ESEM code generator (De Beer and Van Zyl, 2019) was used to generate the Mplus code for ESEM.

### Factorial Validity of the YLPS

In order to examine the factorial validity of the YLPS, the total sample was randomly split into two independent subsamples, subsample A (*n* = 345) and subsample B (*n* = 351), for the purpose of cross-validation.

First, EFA with principal axis factoring extraction and direct oblimin rotation was conducted in subsample A to explore the potential possible number of factors to be extracted.

Second, based on the parallel analysis and scree plots in EFA as well as the theoretical framework, multiple models were constructed and further estimated using ESEM in subsample A. ESEM can be considered as an “integration of the best features of exploratory and confirmatory factor analysis (CFA) within the structural equation modeling framework” (Marsh et al., 2014). One of the main advantages of ESEM is that it allows free cross-loadings of items on multiple factors and therefore provides a less simplistic and more flexible, naturally existing, and valid factor structure (Asparouhov and Muthén, 2009; Marsh et al., 2014). To determine the most appropriate factor structure, the goodness-of-fit values of all candidate models were compared. Several fit indices were evaluated to determine the model fit. Since chi-square tests (χ^2^) are very sensitive to sample size and to minor deviations from multivariate normality, researchers typically focus on sample size-independent indices to assess model fit (Marsh et al., 2005), particularly the comparative fit index (CFI), the Tucker-Lewis index (TLI), the root mean square error of approximation (RMSEA), standardized root mean squared residual (SRMR). CFIs and TLIs greater than 0.900 are considered to present adequate model fit, and values greater than 0.950 are preferable (Hu and Bentler, 1999). RMSEAs smaller than 0.060 and SRMRs smaller than 0.080 support a good model fit (Hu and Bentler, 1999). Moreover, there are some information theoretic indices, such as the Akaike information criterion (AIC), Bayesian information criterion (BIC) and sample-size-adjusted BIC (aBIC), which can be used to compare competing models and make a trade-off between model fit and model complexity. A lower AIC/BIC value indicates a better trade-off between fit and complexity (van de Schoot et al., 2012). In addition, the standardized factor loadings (λ) of each item should also be taken into consideration. Following the recommendations of Osborne et al. (2008), each factor in a model should comprise at least three items with λ greater than 0.320 on their target factor.

Third, after the optimal number of factors and the corresponding ESEM model were obtained, the standardized factor loadings of each item were checked. Items with low loadings (λ < 0.320) on their target factors or substantial cross-loadings (λ > 0.300) were eliminated from the initial 39-item model.

Fourth, the revised model obtained at this stage was cross-validated by the ESEM-within-CFA (when ESEM is estimated with target rotation, it becomes possible to specify *a priori* hypotheses regarding the expected factor structure and thus use ESEM for purely confirmatory purposes; Browne, 2001; Asparouhov and Muthén, 2009) in subsample B, and the goodness-of-fit of the model was checked again. Then, the factor names were assigned based on the revised model. Through the above steps, the final first-order ESEM model was obtained.

However, it is possible that a first-order ESEM model may ignore the presence of hierarchically superior constructs, which will end up being expressed through inflated cross-loadings (Morin et al., 2016). Thus, hierarchical ESEM (H ESEM) and bifactor ESEM (B ESEM) were also conducted according to the suggestion of Morin et al. (2016). In the H ESEM model, all first-order factors were specified as related to a single higher-order factor, with residual correlations among the first-order factors (for a detailed introduction of the H ESEM model, please see Morin et al., 2016). The B ESEM model was estimated in line with typical bifactor assumptions using orthogonal bifactor target rotation. This model partitions the total covariance among the items into a global (G) component underlying all items and specific (S) components explaining the residual covariance not explained by the G factor (for a detailed introduction of B ESEM model, please see Morin et al., 2016). Therefore, in order to choose the best model, we compared the five-factor (first-order) ESEM-within-CFA, H ESEM, B ESEM, and CFA solutions of the YLPS using the total sample to make full use of the data. The analyses in the following steps were all based on the total sample. The same aforementioned criteria were used to determine the most appropriate factor structure. The best model obtained was used for subsequent analyses.

Finally, to test the measurement invariance of the best model, the model was first estimated separately in all gender- and age-related subsamples and then across gender and age groups according to the following steps: (a) configural invariance; (b) weak invariances (loadings); (c) strong invariance (loadings, intercepts); (d) latent means invariance (Meredith, 1993). In each invariance sequence, the preceding model served as a referent. To determine the measurement invariance, the goodness-of-fit values of these models were compared by examining the changes in fit indices in comparison with the preceding more parsimonious model. Following the recommendations of Chen (2007) and Cheung and Rensvold (2002), it is reasonable to select the more complex model over the more parsimonious model when ΔCFI and ΔTLI are greater than 0.010 or decreases in RMSEA are greater than 0.015. The results of the above steps provided empirical support of the factorial validity of the YLPS.

### Reliability and Validity

Subscale composite reliability was computed using McDonald’s (1970)ω = (Σ| λ*_i_*|)^2^/([Σ| λ*_i_*|]^2^ + Σδ*_ii_*), where λ*_i_* are the loadings and δ*_ii_* are the uniquenesses. According to Bagozzi and Yi (1988), composite reliabilities greater than 0.60 are desirable. The latent variable correlations in the final model were evaluated to test the relative independence of the factors.

The common method bias test was carried out before the concurrent validity test. It was evaluated by the model with all factors of the YLPS, CSES, RRSL, and LSI collapsed into one latent factor (Podsakoff et al., 2003). Common method variance was not considered a serious problem if the fit indices indicated a worse fit for the one-factor model.

Then, the latent factor correlations of the YLPS, CSES (problem-focused coping, emotion-focused coping, and social support), RRSL (leadership self-efficacy, leadership flexibility, and task orientation), and LSI (teamwork, self-understanding, communication, decision-making, and fundamentals of leadership) were used to evaluate the concurrent validity of the YLPS. The measurement models for the CSES, RRSL and LSI were specified as CFA factors in accordance with the results of previous validation studies of the constructs (for CSES see Chesney et al., 2006; for RRSL see Chan, 2000; for LSI see Wang et al., 2012), and the measurement model for the YLPS was specified according to the best fitting model obtained in the previous steps. Finally, the criterion-related validity and incremental validity of the YLPS were tested by regression analyses (Boateng et al., 2018).

## Results

### Preliminary Analyses

First, according to Schafer (1999), a missing rate of 5% or less of variables is inconsequential. In the current study, the missing value analysis showed that the missing rates of all items were below 1.5%, and only three items’ missing value rates were above 1%. Second, the high participant-to-item ratio (18:1; Anderson et al., 1992) and good Kaiser–Meyer–Olkin values (KMO = 0.904, *p* < 0.001; Cerny and Kaiser, 1977) suggested that sample size was adequate and the data were suitable for factor analysis.

### Factorial Validity of the YLPS

The theoretical framework suggested a five-factor structure, as described in the literature review section. However, based on the results of EFA, the parallel analysis suggested a four-factor structure, while the scree plot indicated a strong elbow after the fifth factor. Combining these theoretical and empirical considerations, three models with four (Model 1), five (Model 2), and six factors (Model 3), respectively were estimated using ESEM in subsample A. The goodness-of-fit statistics of these three models are displayed in Table 3.

**TABLE 3:** Goodness of fit indices of normal measurement models.

**Model**	**Description**	**Sample**	**χ^2^**	**df**	**CFI**	**TLI**	**RMSEA [90% CI]**	**SRMR**	**AIC**	**BIC**	**aBIC**
**number**											
1	4 first-order factors – 39 items	Subsample A	1019.388	591	0.906	0.882	0.046 [0.041,0.050]	0.038	37854.043	38732.345	38009.055
2	5 first-order factors – 39 items	Subsample A	895.948	556	0.925	0.900	0.042 [0.037,0.047]	0.034	37800.603	38813.733	37979.412
3	6 first-order factors – 39 items	Subsample A	772.236	522	0.945	0.922	0.037 [0.031,0.043]	0.031	37744.891	38888.996	37946.816
4	5 first-order factors – 23 items (ESEM)	Subsample A	351.484	148	0.960	0.932	0.045 [0.035,0.054]	0.027	21610.164	22191.846	21712.826
5	5 first-order factors – 23 items (ESEM-within-CFA)	Subsample B	261.286	148	0.947	0.910	0.047 [0.037,0.056]	0.029	21303.749	21886.728	21407.700
6	5 first-order factors – 23 items (ESEM-within-CFA)	Total sample	319.065	148	0.963	0.937	0.041 [0.035,0.047]	0.022	42646.640	43332.987	42853.534
7	Hierarchical ESEM	Total sample	331.660	153	0.962	0.936	0.041 [0.035,0.047]	0.025	42648.453	43312.074	42848.497
8	Biafactor ESEM	Total sample	239.686	130	0.976	0.954	0.035 [0.028,0.042]	0.019	42603.261	43371.425	42834.819
9	CFA	Total sample	498.914	220	0.940	0.931	0.043 [0.038,0.048]	0.043	42682.489	43041.571	42790.732

*CFI, comparative fit index; df, degrees of freedom; TLI, Tucker–Lewis index; RMSEA, root mean square error of approximation; 90% CI, 90% confidence interval for the RMSEA point estimate; SRMR, standardized root mean squared residual; AIC, akaike information criterion; BIC, bayesian information criterion; aBIC, adjusted bayesian information criterion.*

The results showed unsatisfactory goodness-of-fit indices (TLI < 0.900) for Model 1. Adequate goodness-of-fit indices (CFI and TLI > 0.900; RMSEA < 0.050; SRMR < 0.080) were observed for Models 2 and 3. The indices of Model 3 seemed superior to those of Model 2. However, further investigation suggested that there was a factor in Model 3 comprising less than 3 items with λ greater than 0.32 on their target factor, suggesting that Model 3 might be relatively unstable. Thus, the more parsimonious five-factor model was retained for subsequent analyses.

Based on the standardized factor loadings of the 39-item five-factor model, six items were deleted due to low loading on their target factors, nine items were deleted due to substantial cross-loadings, and 1 item was deleted for both reasons. The resulting 23-item five-factor model of the YLPS was re-estimated by ESEM and provided even better goodness-of-fit indices (see Model 4 in Table 3). The standardized factor loadings of the items in Model 4 are listed in Table 4. The 23-item YLPS showed significant and satisfactory target factor loadings (|0.843 to 0.406|, M|λ| = 0.631; SD| λ| = 0.128) with small cross-loadings. Then, the 23-item five-factor model was cross-validated by ESEM-within-CFA in subsample B and provided satisfactory goodness-of-fit indices (see Model 5 in Table 3). The standardized factor loadings of Model 5 are presented in Table 4.

**TABLE 4:** Exploratory structural equation modeling (ESEM) and ESEM-within-CFA solutions for the YLPS-23.

**Item**	**ESEM (Subsample A, Model 4)**						**ESEM-within-CFA (Subsample B, Model 5)**					
		
	**IN(λ)**	**AT(λ)**	**CO(λ)**	**DE(λ)**	**ST(λ)**	**δ**	**IN(λ)**	**AT(λ)**	**CO(λ)**	**DE(λ)**	**ST(λ)**	**δ**
1	0.063	0.157	**0.635**	–0.011	0.020	0.513	–0.123	0.007	**0.551**	0.111	–0.049	0.654
2	–0.098	0.079	0.013	**0.512**	–0.181	0.760	–0.017	–0.061	0.003	**0.592**	–0.058	0.696
5	–0.003	**0.645**	0.041	–0.051	–0.051	0.609	0.038	**0.560**	0.016	0.109	–0.118	0.673
8	0.038	–0.073	0.212	**0.509**	0.112	0.554	–0.023	0.007	–0.036	**0.657**	0.076	0.536
9	–0.003	0.049	0.129	0.001	**0.584**	0.574	–0.009	0.073	–0.117	0.216	**0.539**	0.564
10	–0.006	–0.105	0.124	–0.033	**0.767**	0.427	–0.067	–0.023	0.020	0.038	**0.711**	0.461
11	**0.656**	0.045	–0.119	0.049	0.034	0.563	**0.584**	0.164	–0.112	–0.036	0.046	0.656
12	0.032	0.158	0.121	**0.433**	0.050	0.642	0.062	0.032	0.080	**0.361**	0.153	0.734
16	0.017	–0.021	–0.006	**0.708**	0.012	0.500	0.059	0.091	0.150	**0.442**	0.076	0.627
17	–0.035	–0.014	**0.671**	0.039	0.102	0.481	0.022	–0.084	**0.882**	–0.031	0.032	0.258
21	0.005	0.007	**0.406**	0.149	–0.249	0.779	0.113	0.145	**0.405**	0.070	–0.136	0.761
23	–0.090	**0.489**	0.020	0.054	0.088	0.670	–0.209	**0.366**	0.122	0.076	0.107	0.595
24	**0.805**	–0.004	0.048	–0.037	–0.015	0.352	**0.767**	–0.023	0.015	–0.090	0.054	0.424
26	–0.062	0.115	**0.455**	0.087	–0.054	0.720	0.054	0.148	**0.416**	–0.019	0.142	0.699
27	–0.032	0.098	–0.064	0.016	**0.761**	0.360	–0.049	–0.049	0.044	–0.111	**0.902**	0.261
28	–0.027	0.027	–0.017	0.128	**0.718**	0.379	0.014	0.014	0.035	0.017	**0.747**	0.404
31	**0.734**	–0.080	0.041	–0.031	–0.017	0.439	**0.614**	–0.041	–0.009	0.090	–0.060	0.595
33	–0.011	**0.693**	0.074	–0.004	0.069	0.441	–0.064	**0.663**	–0.013	–0.056	0.012	0.570
34	0.019	**0.711**	–0.061	0.008	0.012	0.507	0.073	**0.680**	0.017	–0.057	0.046	0.530
35	**0.843**	0.108	–0.013	0.020	–0.010	0.294	**0.679**	–0.070	0.071	0.073	–0.039	0.497
36	**0.669**	–0.056	0.082	–0.035	–0.012	0.538	**0.640**	–0.058	0.090	–0.043	–0.010	0.574
38	**0.661**	–0.013	–0.115	0.158	–0.001	0.513	**0.520**	–0.033	–0.045	0.067	0.015	0.719
39	0.111	0.187	0.008	0.081	**0.441**	0.659	0.189	0.052	–0.006	0.079	**0.491**	0.665

*The main loadings of the items onto their target factor are in bold and cross-loadings are in regular font. CFA, confirmatory factor analysis; ESEM, exploratory structural equation modeling; IN, leadership information; AT, leadership attitude; CO, communication skills; DE, decision-making skills; ST, stress management skills; λ, factor loading; δ, uniquenesses.*

Specifically, the first factor comprises 6 items with significant target loadings (i.e., items 11, 24, 31, 35, 36, and 38), all reflecting leadership information. The second factor contains 4 items with significant target loadings (i.e., items 5, 23, 33, and 34), all reflecting leadership attitude. The third factor comprises four items with significant target loadings (i.e., items 1, 17, 21, and 26), all reflecting communication skills. The fourth factor contains 4 items with significant target loadings (i.e., 2, 8, 12, and 16), all reflecting decision-making skills. The fifth factor contains five items with significant target loadings (i.e., 9, 10, 27, 28, and 39), all reflecting stress management skills.

The goodness-of-fit indexes of the first-order ESEM-within-CFA model with five factors, H ESEM, B ESEM and CFA are presented in Table 3 (Model 6–9). The fitness is better for Models 6 and 8 than for Models 7 and 9.

To further compare Models 6 and 8, the factor loadings of these two models are reported in Table 5. The target loadings of the G factor in Model 8 are not high, and many are lower than 0.32 (|0.020 to 0.571|, M|λ| = 0.331, SD|λ| = 0.179) and the target loadings of the S factor (leadership information: |0.604 to 0.798|, M|λ| = 0.690, SD|λ| = 0.084; leadership attitude: |0.329 to 0.610|, M|λ| = 0.503, SD|λ| = 0.123; communication skills: |0.424 to 0.568|, M|λ| = 0.464, SD|λ| = 0.070; decision-making skills: |0.294 to 0.500|, M|λ| = 0.397, SD|λ| = 0.088; stress management skills: |0.336 to 0.635|, M|λ| = 0.448, SD|λ| = 0.138) is not as good as that of Model 6 (leadership information: |0.620 to 0.792|, M|λ| = 0.688, SD|λ| = 0.079; leadership attitude: |0.437 to 0.695|, M|λ| = 0.609, SD|λ| = 0.120; communication skills: |0.425 to 0.745|, M|λ| = 0.566, SD|λ| = 0.140; decision-making skills: |0.409 to 0.627|, M|λ| = 0.548, SD|λ| = 0.098; and stress management skills: |0.474 to 0.828|, M|λ| = 0.678, SD |λ| = 0.145). Therefore, the five-factor (first-order) ESEM -within-CFA model (Model 6) was considered the best model and retained in the following analysis.

**TABLE 5:** Exploratory structural equation modeling (ESEM)-within-CFA and B ESEM solutions for the YLPS-23.

**Item**	**ESEM-within-CFA (Model 6)**						**B ESEM (Model 8)**						
		
	**IN(λ)**	**AT(λ)**	**CO(λ)**	**DE(λ)**	**ST(λ)**	**δ**	**G(λ)**	**IN(λ)**	**AT(λ)**	**CO(λ)**	**DE(λ)**	**ST(λ)**	**δ**
1	–0.019	0.054	**0.605**	0.021	0.004	0.597	**0.468**	0.008	0.008	**0.432**	–0.006	–0.097	0.585
2	–0.076	–0.008	–0.045	**0.627**	–0.127	0.698	**0.326**	0.015	–0.028	–0.001	**0.429**	–0.105	0.698
5	0.015	**0.614**	0.003	0.064	–0.099	0.636	**0.300**	–0.013	**0.514**	0.034	0.051	–0.042	0.640
8	0.012	–0.057	0.066	**0.550**	0.112	0.606	**0.510**	0.088	–0.091	0.041	**0.364**	0.028	0.589
9	–0.009	0.046	–0.014	0.088	**0.583**	0.587	**0.571**	–0.023	–0.021	–0.118	–0.017	**0.336**	0.546
10	–0.030	–0.076	0.063	–0.028	**0.760**	0.445	**0.560**	–0.060	–0.102	–0.044	–0.076	**0.488**	0.426
11	**0.620**	0.101	–0.126	0.014	0.031	0.618	−**0.062**	**0.604**	0.105	–0.033	0.082	0.079	0.606
12	0.039	0.073	0.069	**0.409**	0.112	0.698	**0.435**	0.087	0.052	0.085	**0.294**	0.082	0.700
16	0.032	0.010	0.028	**0.606**	0.045	0.575	**0.403**	0.108	0.033	0.117	**0.500**	0.095	0.552
17	–0.003	–0.071	**0.745**	–0.008	0.081	0.440	**0.469**	0.030	–0.062	**0.568**	0.026	0.005	0.452
21	0.053	0.034	**0.425**	0.109	–0.198	0.780	**0.142**	0.089	0.067	**0.431**	0.157	–0.095	0.748
23	–0.127	**0.437**	0.069	0.019	0.117	0.688	**0.441**	–0.152	**0.329**	–0.004	–0.046	0.030	0.671
24	**0.792**	–0.010	0.032	–0.056	0.008	0.381	−**0.095**	**0.779**	0.008	0.092	0.045	0.048	0.371
26	–0.007	0.093	**0.490**	–0.001	0.041	0.702	**0.344**	0.003	0.096	**0.424**	0.044	0.036	0.689
27	–0.043	0.015	–0.026	–0.030	**0.828**	0.332	**0.520**	–0.086	0.028	–0.046	–0.016	**0.616**	0.340
28	–0.003	0.017	–0.008	0.064	**0.744**	0.392	**0.481**	–0.033	0.061	0.032	0.103	**0.635**	0.349
31	**0.681**	–0.077	0.018	0.025	–0.034	0.508	−**0.132**	**0.687**	–0.035	0.104	0.115	0.041	0.484
33	–0.021	**0.695**	0.037	–0.061	0.037	0.501	**0.428**	–0.075	**0.560**	–0.003	–0.081	0.002	0.492
34	0.048	**0.688**	–0.021	–0.003	0.017	0.532	**0.323**	–0.003	**0.610**	0.037	0.034	0.080	0.515
35	**0.771**	0.030	0.022	0.009	–0.010	0.404	**0.129**	**0.798**	–0.060	–0.069	–0.052	–0.141	0.316
36	**0.668**	–0.050	0.109	–0.083	0.003	0.547	**0.040**	**0.668**	–0.100	0.033	–0.082	–0.091	0.526
38	**0.596**	–0.031	–0.074	0.101	0.017	0.620	**0.020**	**0.604**	–0.051	–0.051	0.083	–0.012	0.622
39	0.142	0.115	0.004	0.061	**0.474**	0.673	**0.404**	0.120	0.107	0.013	0.064	**0.366**	0.673

*The main loadings of the items onto their target factor are in bold and cross-loadings are in regular font. ESEM, exploratory structural equation modeling; B ESEM, bifactor exploratory structural equation modeling; IN, leadership information; AT, leadership attitude; CO, communication skills; DE, decision skills; ST, stress management skills; G, global factor; λ, factor loading; δ, uniquenesses.*

### Measurement Invariance

The measurement invariance of the final five-factor (first-order) ESEM-within-CFA model was tested. Two ESEM-within-CFA models were conducted for female subsample (CFI = 0.956, TLI = 0.925, RMSEA = 0.047; SRMR = 0.028), and male subsample (CFI = 0.964, TLI = 0.939, RMSEA = 0.038; SRMR = 0.029), separately (Model 10 in Table 6). In different gender subsamples, complete invariance (loading, intercepts, and latent means; Models 11–14 in Table 6) was achieved with small changes in the fit indices (ΔCFIs/TLIs ≤ 0.010, ΔRMSEAs ≤ 0.015) and Model 14 has the lowest BIC value, confirming the equivalence of the 5 first-order factors model of the YLPS across gender.

**TABLE 6:** Goodness of fit indices of measurement invariance models.

**Model**	**Description**	**Sample**	**χ^2^**	**df**	**CFI**	**TLI**	**RMSEA [90% CI]**	**SRMR**	**AIC**	**BIC**	**aBIC**
**number**											
10	5 first-order factors – 23 items	Female (*n* = 377)	269.054	148	0.956	0.925	0.047 [0.038,0.055]	0.028	22340.581	22934.350	22455.263
	5 first-order factors – 23 items	Male (*n* = 319)	216.892	148	0.964	0.939	0.038 [0.027,0.049]	0.029	20234.853	20803.397	20324.455
11	Gender-configural	Total sample	485.947	296	0.960	0.931	0.043 [0.036,0.050]	0.028	42575.434	43948.130	42989.224
12	Gender-weak	Total sample	621.136	386	0.950	0.934	0.042 [0.036,0.048]	0.043	42530.623	43494.237	42821.098
13	Gender-strong	Total sample	654.564	404	0.947	0.933	0.042 [0.036,0.048]	0.044	42528.051	43409.849	42793.863
14	Gender-latent mean	Total sample	676.045	409	0.943	0.930	0.043 [0.037,0.049]	0.049	42539.532	43398.603	42798.493
15	5 first-order factors – 23 items	Seventh grade (*n* = 140)	187.312	148	0.969	0.947	0.044 [0.020,0.062]	0.034	8592.358	9036.546	8558.802
	5 first-order factors – 23 items	Eighth grade (*n* = 203)	200.232	148	0.953	0.920	0.042 [0.025,0.056]	0.033	13106.341	13606.636	13128.230
	5 first-order factors – 23 items	Ninth grade (*n* = 353)	284.260	148	0.944	0.905	0.051 [0.042,0.060]	0.029	20997.064	21580.901	21101.868
16	Grade-configural	Total sample	671.804	444	0.953	0.919	0.047 [0.040,0.054]	0.032	42695.764	44754.807	43316.449
17	Grade-partial weak	Total sample	706.357	484	0.954	0.928	0.044 [0.037,0.051]	0.037	42650.317	44527.547	43216.196
18	Grade-partial strong	Total sample	740.206	518	0.954	0.932	0.043 [0.036,0.050]	0.039	42616.166	44338.854	43135.459
19	Grade-partial latent mean	Total sample	779.455	528	0.948	0.925	0.045 [0.038,0.052]	0.047	42635.415	44312.649	43141.006

*CFI, comparative fit index; df, degrees of freedom; TLI, Tucker–Lewis index; RMSEA, root mean square error of approximation; 90% CI, 90% confidence interval for the RMSEA point estimate; SRMR, standardized root mean squared residual; AIC, Akaike information criterion; BIC, Bayesian information criterion; aBIC, adjusted Bayesian information criterion.*

Three ESEM-within-CFA models were conducted for seventh grade subsample (CFI = 0.969, TLI = 0.947, RMSEA = 0.044, SRMR = 0.034), eighth grade subsample (CFI = 0.953, TLI = 0.920, RMSEA = 0.042, SRMR = 0.033), and ninth grade subsample (CFI = 0.944, TLI = 0.905, RMSEA = 0.051, SRMR = 0.029), separately (Model 15 in Table 6). In different grade/age subsamples, the configural model (Model 16 in Table 6) achieved a satisfactory fitness of the data (CFI = 0.953, TLI = 0.919, RMSEA = 0.047, SRMR = 0.032), supporting the configural invariance across grade/age subsamples. A partial weak invariance (Model 17 in Table 6) was supported after releasing equality constraints of loadings of item 26 (i.e., “I am good at recognizing non-verbal signs from others.”). In addition, partial strong invariance (Model 18 in Table 6) and partial latent means invariance (Model 19 in Table 6) were achieved across grade/age subsamples after the invariance constraints were released for item 26, and Model 19 has the lowest BIC value. These results indicated that students differing in grade/age might have different understandings of item 26. Generally speaking, the first-order model with five factors demonstrated good measurement invariance across different groups.

### Composite Reliability and Inter-Factor Correlations

All factors presented acceptable to modest composite reliability coefficients (from ω = 0.651 for the decision-making skills dimension to ω = 0.847 for the leadership information dimension; see Table 7). In addition, the latent variable correlations were small to moderate, confirming the relative independence of the dimensions.

**TABLE 7:** Composite reliability and inter-factor correlations.

**Correlations**	**IN**	**AT**	**CO**	**DE**	**ST**
IN	(0.847)				
AT	−0.099*	(0.715)			
CO	0.051	0.338***	(0.671)		
DE	0.155**	0.341***	0.510***	(0.651)	
ST	–0.076	0.397***	0.332***	0.453***	(0.825)

*p < 0.05, **p < 0.01, ***p < 0.001; The values on the diagonal are the composite reliability of the dimensions. IN, leadership information; AT, leadership attitude; CO, communication skills; DE, decision-making skills; ST, stress management skills.

### Common Method Bias

The fit indices indicated a worse fit for the one-factor model (CFI = 0.564, TLI = 0.551, RMSEA = 0.072), which suggested that common method variance was not a serious problem.

### Concurrent Validity

The latent variables correlations are reported in Table 8. The five dimensions of the YLPS had significant positive correlations with almost all dimensions of the three concurrent scales, i.e., CSES, RRSL and LSI. For example, the decision-making dimension of LSI was highly correlated with the decision-making skill dimension of the YLPS as expected (*r* = 0.646, *p* < 0.001), and the leadership self-efficacy dimension of RRSL was highly correlated with the leadership attitude dimension of the YLPS as expected (*r* = 0.666, *p* < 0.001). These results provide evidence for the good concurrent validity of the YLPS.

**TABLE 8:** Concurrent validity of the YLPS.

	**Dimensions of YLPS**				
	
	**LI**	**LA**	**CS**	**DM**	**SM**
CSES1	0.06	0.360***	0.245***	0.397***	0.703***
CSES2	0.005	0.291***	0.178***	0.172**	0.587***
CSES3	0.186***	0.404***	0.326***	0.307***	0.500***
RRSL1	–0.014	0.666***	0.403***	0.315***	0.536***
RRSL2	0.181***	0.651***	0.386***	0.427***	0.434***
RRSL3	0.190***	0.471***	0.499***	0.500***	0.498***
LSI1	0.292***	0.441***	0.387***	0.452***	0.539***
LSI2	0.241***	0.454***	0.302***	0.401***	0.618***
LSI3	0.117*	0.596***	0.441***	0.444***	0.587***
LSI4	0.231***	0.411***	0.409***	0.646***	0.490***
LSI5	−0.096*	0.747***	0.391***	0.227***	0.459***

*p < 0.05, **p < 0.01, ***p < 0.001; LI, leadership information; LA, leadership attitude; CS, communication skills; DM, decision-making skills; SM, stress management skills; CSES1, problem-focused coping; CSES2, emotion-focused coping; CSES3, social support; RRSL1, leadership self-efficacy; RRSL2, leadership flexibility; RRSL3, task orientation; LSI1, teamwork; LSI2, self-understanding; LSI3, communication; LSI4, decision-making; LSI5, fundamentals of leadership.

### Criterion-Related Validity and Incremental Validity

The length of time participants served as class leaders was treated as the dependent variable, and the latent factors of the YLPS were treated as independent variables. The results of the regression analysis were reported in Table 9. According to Model c, leadership information, leadership attitude, and communication skills could significantly predict the criterion. The comparison between Models a and b suggested that after controlling for the effects of gender, age and family SES, the five latent factors could still explain 6.2% of the variation. These results provided evidence of the criterion-related validity of the YLPS. The comparison between Models d and e suggested that the five latent factors could explain 7.7% of the variation over and above the CSES, RRSL and LSI, demonstrating the incremental validity of the YLPS. The results of the regression analysis also suggested that the length of leadership position was negatively predicted by gender but positively predicted by grade/age and family SES.

**TABLE 9:** Criterion-related validity of the YLPS and the incremental validity of the YLPS over CSES, RRSL, and LSI.

**Length of**	**Model**									
	
**leadership**										
**positions**										
	**a**		**b**		**c**		**d**		**e**	
					
	***b*(SE)**	**β**	***b*(SE)**	**β**	***b*(SE)**	**β**	***b*(SE)**	**β**	***b*(SE)**	**β**
Gender	−1.07(0.22)	–0.19***	−1.08(0.21)	–0.19***						
Grade	0.54 (0.14)	0.15***	0.60 (0.14)	0.17***						
SES	0.50 (0.11)	0.18***	0.41 (0.11)	0.15***						
LI			0.35 (0.13)	0.13**	0.46 (0.13)	0.17***			0.44 (0.47)	0.16
LA			0.55 (0.15)	0.20***	0.39 (0.16)	0.14*			−0.52(3.64)	–0.18
CS			0.06 (0.15)	0.02	0.46 (0.19)	0.17*			0.48 (1.41)	0.17
DM			−0.12(0.19)	–0.04	−0.24(0.22)	–0.09			−0.21(1.41)	–0.08
SM			0.23 (0.14)	0.08	0.20 (0.15)	0.07			0.44 (3.11)	0.16
CSES1							−0.03(1.59)	–0.02	0.29 (2.99)	0.18
CSES2							−0.13(0.46)	–0.09	−0.15(0.44)	–0.10
CSES3							0.01 (2.08)	0.01	−0.53(3.50)	–0.30
RRSL1							2.39 (10.57)	0.61	3.91 (8.02)	0.97
RRSL2							−1.29(1.91)	–0.27	1.52 (13.67)	0.31
RRSL3							−0.21(10.47)	–0.05	−4.20(6.81)	–0.90
LSI1							2.89 (24.13)	0.65	9.05 (13.18)	2.06
LSI2							−2.23(21.61)	–0.34	−8.11(6.22)	–1.23
LSI3							−0.62(9.44)	–0.15	−4.06(3.58)	–0.99
LSI4							−0.36(2.70)	–0.08	0.04 (7.65)	0.01
LSI5							0.03 (1.75)	0.00	2.39 (16.62)	0.27
Constant	3.41 (0.36)		3.28 (0.35)		4.09 (0.11)		4.08 (0.11)		4.08 (0.11)	
*R*^2^	0.099		0.161		0.083		0.087		0.164	

*p < 0.05, **p < 0.01, ***p < 0.001; LI, leadership information; LA, leadership attitude; CS, communication skills; DM, decision-making skills; SM, stress management skills; CSES1, problem-focused coping; CSES2, emotion-focused coping; CSES3, social support; RRSL1, leadership self-efficacy; RRSL2, leadership flexibility; RRSL3, task orientation; LSI1, teamwork; LSI2, self-understanding; LSI3, communication; LSI4, decision-making; LSI5, fundamentals of leadership.

## Discussion

Emphasizing the importance of leadership development at an early age, the development theory of leadership has attracted attention from both researchers and practitioners for many years (Ricketts and Rudd, 2002; Anderson, 2009). This theory has a strong theoretical basis (Linden and Fertman, 1998) and has gained support in recent research (Bruce and Stephens, 2017). However, no attempt has been made to develop a youth leadership scale based on the theory. Existing youth leadership evaluation scales mainly focus on the skills that adolescents have acquired and demonstrated. The development theory proposes that in addition to leadership skills, two other aspects should be considered when assessing young people’s leadership potential. One is leadership knowledge and information, and the other is the attitude toward being a leader. Young people who accumulate the necessary knowledge and information about leadership, develop a positive attitude toward leadership, and demonstrate key leadership skills are more likely to grow up to become leaders. That is, they are adolescents with high leadership potential. To facilitate the research and practice of youth leadership development, the current study constructed a youth leadership potential scale based on the development theory of leadership.

The results of the current study suggest that the psychometric properties of the YLPS are satisfactory. First, the results of the ESEM in subsample A and ESEM-within-CFA in subsample B cross-validated the first-order five-dimensional structure of the scale. The five dimensions include leadership information, leadership attitude and three leadership skills, i.e., communication skills, decision-making skills and stress management skills. The rationality of the first-order five-dimensional ESEM model was further confirmed when compared with the H ESEM, B ESEM, and CFA models. Results suggested that the first-order ESEM-within-CFA model fitted the data better than the others, which is in accordance with the development theory of leadership. According to the theory, the five dimensions of the YLPS are relatively distinctive and representing different aspects of youth leadership potential (Linden and Fertman, 1998). The first-order ESEM-within-CFA model specifies five unique factors and allows for cross-loadings. However, the H ESEM model specifies all first-order factors as related to a single higher-order factor and the B ESEM model uses the G factor and S factor together to represent the total covariance among all items, both of which specify a global level of youth leadership potential and influence the expression of dimensional uniqueness to some extent (Fadda et al., 2017). That is why the less restrictive ESEM within CFA models is the best one. In addition, the measurement invariance test suggested that the first-order five-dimensional structure of the scale generally performed well in different gender groups and grade/age groups.

Second, the acceptable-to-modest composite reliability of each dimension and the small-to-moderate latent variable correlations among the dimensions in the final model suggested that the YLPS is reliable and the five dimensions are relatively independent. Third, the five dimensions of the YLPS were significantly positively correlated with almost all dimensions of three other commonly used scales assessing youth leadership, suggesting its sufficient concurrent validity. Finally, regression analysis suggested that the YLPS could predict the variation in the length of time students serve as class leaders over and above both major demographic variables (gender, age, and family SES) and existing youth leadership scales (i.e., CSES, RRSL, and LSI), thus providing evidence of the YLPS’s criterion-related validity and incremental validity. In sum, the current study concluded that the 23-item five-dimensional YLPS is a promising tool for assessing the leadership potential of adolescents.

The development of the YLPS has both theoretical and practical implications. With regard to the theoretical aspect, constructing a scale based on the development theory of leadership should spur future empirical studies to explore the mystery of leadership from a developmental perspective. Extant research on the development theory of leadership is mainly theoretical and conceptual (Ricketts and Rudd, 2002), largely because there is no scale based on this theory. The timely development of the YLPS will fill this gap. We call for more empirical studies to test and enrich the development theory of leadership. According to the development theory of leadership, the development of youth leadership can be divided into three stages: awareness, interaction, and mastery (Linden and Fertman, 1998). Before the *awareness stage*, adolescents generally are unaware that they are leaders, and leadership seems to be distant from them. With the accumulation of information on a variety of leaders and ways to lead, they begin the process of recognizing and identifying their leadership potential. After the awareness stage, adolescents begin to see themselves as leaders and actively strengthen and broaden their leadership potential and skills through interactions with others and their social environment. They are now in the *interaction stage*, in which they receive feedback through their interactions. As a result, their confidence as leaders increases, and their leadership skills improve considerably. With the skills and experience they have acquired in the interaction stage, adolescents enter the *mastery stage*, where they gradually become mature leaders and influence others. Future longitudinal studies could utilize the YLPS to testify the three-stage conceptual model and trace the development of youth leadership along different dimensions in each stage.

In terms of practical implications, because the YLPS simultaneously measures the leadership knowledge and information dimension, the leadership attitude, will, and desire dimension and the leadership skills dimension, it provides a comprehensive measurement framework. In leadership training practice, the YLPS can be used in screening programs for youth leadership potential. Based on the screening results on different dimensions of the YLPS, educators and trainers can provide more effective programs targeting those aspects of leadership potential that need more support. Adolescents’ leadership potential can be cultivated more efficiently when a leadership development curriculum is developed based on individual differences in the different dimensions of leadership potential. For example, if Jerry scored high on most leadership skills but low on leadership attitude while Tom scored the opposite, it would be inefficient for them to go through the same leadership developing program. The YLPS makes it possible to design a training program and select training content according to individual needs. However, it is important to note the potential negative aspect of youth leadership potential gauging. Low scores might hurt young people and block their path to growing into a great leader. Thus, using the YLPS in practice should focus on nurturing youth leadership development in different aspects rather than dividing youth into different levels.

This study found interesting results on the relationship between demographic variables and YLPS scores. The results suggested that the length of leadership positions since primary school was longer for girls than for boys. Similarly, related studies have found that girls are more likely to be nominated as leaders (Waasdorp et al., 2013). However, this finding is opposite to our observations that males rather than females dominate leadership positions in adulthood (Bear et al., 2017). It would be interesting for future studies to address several questions: What happens to girls during the process of growing up that hinders them from benefiting from their leadership experience that they develop at an early age? What is the turning point? Is this phenomenon mainly due to generally held stereotypes toward female leaders, or is it possibly due to insufficient development of some dimensions of leadership potential? Another possibility is that it is simply due to cultural perceptions (Albirini, 2006). That is, even if women have equal or sometimes even higher leadership potential, they are less likely to be selected as leaders because of common beliefs and values in society. In addition, the results also showed that family SES is positively associated with the length of leadership positions. This finding is consistent with previous research. For example, Li et al. (2011) found that individuals from high-SES families are more likely to assume leadership positions in adulthood. It would be beneficial to explore how family SES influences the development of different dimensions of youth leadership potential and the underlying mechanism of those effects. These are all important questions that could be answered in future research.

The limitations of the current study call for researchers to collect further evidence of the reliability and validity of the YLPS. First, given that the current study is cross-sectional, the test–retest reliability of the scale cannot be established. Second, all measures in the current study are self-rated. Although data analysis suggests that common method bias is not a serious problem, assessment accuracy is a concern that cannot be ignored. That is, cognitive or motivational bias might impede youth in accurately assessing themselves. Actually, some youth leadership assessment tools are teacher-rated. However, researchers have suggested that teachers’ evaluation of students may be influenced by students’ past performance, which is an indicator of halo bias creeping into the assessment process, as high-performance scores tend to be generalized to other characteristics, often incorrectly so (Dries and Pepermans, 2012). Future studies could use multisource data to corroborate each other. Third, the participants in the current study came from one middle school. As shown in Table 2, gender was roughly balanced, and the parents’ highest educational degree obtained was diverse. In a supplementary analysis, we tested the normality of family SES using the skewness and kurtosis of the distribution. The results suggested that the distribution of family SES (skewness = −1.050, kurtosis = 1.029) was not substantially differ from normality (West et al., 1995). These results provide some evidence of the representativeness of our sample. However, future research based on a more diversified sample would be helpful to further verify the generalizability of the results. Fourth, in the current study, we chose the length of time participants served as class leaders as the criterion to test the criterion-related validity of the YLPS. Besides the length of leadership, leadership effectiveness could also be evaluated in future study as a criterion to support the validity of the scale. Given that youth leadership potential is inherently future-oriented, research using a longitudinal design would be helpful to provide further evidence of the predictive validity of the YLPS.

The development theory of leadership is a proactive theory because it emphasizes that leadership can be learned, cultivated and nurtured. Adding leadership knowledge and attitude to the youth leadership potential model can allow researchers and practitioners to focus more on the acquirability of leadership. Such a developmental leadership perspective might improve adolescents’ leadership self-efficacy, which may have many positive developmental effects (Murphy and Johnson, 2011). Future research would be helpful to explore this possibility. In addition, in the current study, we strictly followed the dimensions suggested by Linden and Fertman (1998); future research is needed to explore whether there are other fundamental leadership skills that should be added to update the model.

## Data Availability Statement

The datasets generated for this study are available on request to the corresponding author.

## Ethics Statement

The studies involving human participants were reviewed and approved by Committee on Human Protection and Ethics in Psychology at Beijing Normal University. Written informed consent to participate in this study was provided by the participants’ legal guardian/next of kin.

## Author Contributions

YY and XS developed the study concept, created the study design, and drafted the manuscript. QC performed testing and data collection. YY and ZL performed the data analysis and interpretation. GX and DY provided revisions. All authors approved the final version of the manuscript for submission.

## Conflict of Interest

QC was employed by the Yunnan Provincial Natural Gas Co., Ltd. The remaining authors declare that the research was conducted in the absence of any commercial or financial relationships that could be construed as a potential conflict of interest.
